# Supplementary material for: A Vaccine Construction against COVID-19-Associated Mucormycosis Contrived with Immunoinformatics-Based Scavenging of Potential Mucoralean Epitopes
Source: Vaccines (Basel). 2022 Apr 22;10(5):664. doi: 10.3390/vaccines10050664 (PMC9147184; doi:10.3390/vaccines10050664)
Supplement: Supplementary file 1 [file vaccines-10-00664-s001.zip › vaccines-1673194-supplementary.pdf]

Table S1: Default parameters of the utilized tools for epitope prediction.

Tool Used	Default Parameters
CELLO2GO	E-value = 0.001
Vaxijen 2.0	Threshold = 0.5
Bepipred 2.0 (phytoene dehydrogenase)	Threshold = 0.5
Bepipred 2.0 (hypothetical protein)	Threshold = 0.5
Surface accessibility (phytoene dehydrogenase)	1.000
Surface accessibility (hypothetical protein)	1.000
Antigenicity analysis (phytoene dehydrogenase)	1.027
Antigenicity analysis (hypothetical protein)	1.014
Flexibility analysis (phytoene dehydrogenase)	0.990
Flexibility analysis (hypothetical protein)	0.998
B-turn analysis (phytoene dehydrogenase)	0.967
B-turn analysis (hypothetical protein)	1.014
Hydrophilicity analysis (phytoene dehydrogenase)	1.649
Ellipro	Minimum score = 0.5<br>Maximum distance in angstroms = 6
MHCI prediction	Sort peptides = descending score<br>Output = XHTML table
MHCII prediction	Sort peptides = Adjusted rank<br>Output = XHTML table

Table S2: Linear epitopes of the target proteins predicted by Bepipred 2.0.

No.	Start	End	Peptide	Length
Phytoene dehydrogenase				
1	73	84	DERIQDHLELLR	12
2	114	122	RVEGPLGFG	9
3	144	153	KKNFESIWDL	10
4	155	155	R	1
5	157	159	KYA	3
6	179	180	FK	2
7	217	217	G	1
8	292	305	LPPCRWTQNTLASK	14
9	321	325	KVPQL	5
10	337	350	QESFDEIFKDFGLP	14
11	364	370	DPSAAPD	7
12	384	402	MKSKTGDASTENYPAMVDK	19
13	415	423	LGMSNFADL	9
14	429	430	VN	2
15	436	436	Q	1
16	460	469	RPSTKDSTGR	10
17	504	530	FGKTPKPRKIEMENTQAPLEEPDAEST	27
18	555	561	NGQTPAS	7
19	572	576	RVHNS	5
Hypothetical protein				
1	32	42	KIYKKTTKKHQ	11
2	76	76	S	1
3	83	96	MNAEEPSAAQMFNP	14
4	126	141	PSKKSKTTKKVRLMNG	16
5	173	177	DNSTT	5
6	215	221	GSTPESG	7
7	241	255	TEGEALDALGLKRYC	15

Table S3: Predicted peptides based on the surface accessibility scores.

No.	Start	End	Peptide	Length
Phytoene dehydrogenase				
1	48	56	QGHRFDQGP	9
2	72	77	LDERIQ	6
3	86	92	DNNYKVH	7
4	104	111	DLTRMKAE	8
5	128	134	MKETHIH	7
6	171	184	IYDRASKYFKTKKM	14
7	235	241	IAKQKYD	7
8	253	261	INTDDATKQ	9
9	297	303	WTQNTLA	7
10	384	396	MKSKTGDASTENY	13
11	460	471	RPSTKDSTGRYD	12
12	504	529	FGKTPKPRKIEMENTQAPLEEPDAES	26
13	552	559	PQSNGQTP	8
Hypothetical protein				
1	31	41	DKIYKKTTKKH	11
2	50	55	FRPKKS	6
3	83	89	MNAEEPS	7
4	125	135	PPSKKSKTTKK	11
5	171	176	DFDNST	6
6	198	205	LETYEMDE	8

Table S4: Predicted epitopes for both proteins based on antigenicity analysis.

No.	Start	End	Peptide	Length
Phytoene dehydrogenase				
1	4	11	KHIVIIGA	8
2	42	48	CSLIHHQ	7
3	56	62	PSLYLMP	7
4	79	85	HLELLRC	7
5	159	170	APEIFRLHLFGK	12
6	201	212	DAPAVYSLLQYT	12
7	226	235	NMVVQKLEAI	10
8	247	252	NAPVAK	6
9	274	296	DADAVVCNADLVYAYHNLLPPCR	23
10	309	314	SSSISF	6
11	322	335	VPQLDVHNIFLAEA	14
12	352	360	EASFYVNVP	9
13	374	382	SVIVLVPIG	9
14	405	413	KMVLAVIER	9
15	446	459	ILGLSHDVLQVLWF	14
16	473	479	LFFVGAS	7
17	484	493	TGVPIVLAGS	10
18	497	503	SDQVVKS	7
19	530	536	TFPVWFW	7
20	567	573	LPEVFRV	7
Hypothetical protein				
1	22	28	CVNIIDL	7
2	42	51	QTICMAVKFR	10
3	96	105	PPFVYSLAIS	10
4	120	126	IQLISPP	7
5	145	152	MVNCLSFI	8
6	154	160	PNKLLSG	7
7	165	170	KLAVWS	6
8	206	215	KVYIAAAGVG	10
9	232	238	YLSLQQA	7
10	246	274	LDALGLKRYCCRRMVLTHVDLIEKLLHYN	29

Table S5: MHC-I binding epitopes selected from phytoene dehydrogenase**.**

Allele	Start	End	Peptide	IC50
HLA-A*32:01	539	547	AAFWVMFMF	497.49
HLA-A*02:06	539	547		346.37
HLA-A*23:01	539	547		254.76
HLA-A*30:02	539	547		199.03
HLA-B*35:01	539	547		115.97
HLA-B*57:01	539	547		101.52
HLA-B*58:01	539	547		43.91
HLA-B*15:01	170	178	KIYDRASKY	482.96
HLA-B*15:02	170	178		477.82
HLA-A*11:01	170	178		150.05
HLA-C*14:02	170	178		121.42
HLA-A*30:02	170	178		49.72
HLA-A*03:01	170	178		27.33
HLA-B*15:01	310	318	SSISFYWSM	467.25
HLA-B*57:01	310	318		444.12
HLA-A*68:02	310	318		266.14
HLA-A*26:01	310	318		169.37
HLA-A*02:06	310	318		148.3
HLA-A*32:01	310	318		137.42
HLA-B*58:01	310	318		100.09
HLA-C*07:01	186	194	MAFTFQTMY	476.37
HLA-B*15:01	186	194		394.55
HLA-A*03:01	186	194		388.7
HLA-A*11:01	186	194		229.42
HLA-B*53:01	186	194		160.91
HLA-A*30:02	186	194		45.69
HLA-B*58:01	186	194		27.21
HLA-A*68:01	186	194		16.17
HLA-B*57:01	186	194		15.83
HLA-B*35:01	186	194		5.74
HLA-B*58:01	529	537	STFPVWFWL	396.56
HLA-B*57:01	529	537		126.8
HLA-A*31:01	529	537		93.11

HLA-A*02:01	529	537		58.03
HLA-A*32:01	529	537		27.46
HLA-A*02:06	529	537		9.15
HLA-A*68:02	529	537		5.07
HLA-A*02:01	543	551	VMFMFFYFF	307.29
HLA-A*02:06	543	551		234.63
HLA-A*30:02	543	551		95.92
HLA-A*31:01	543	551		66.2
HLA-A*24:02	543	551		56.46
HLA-B*15:01	543	551		40.17
HLA-A*23:01	543	551		33.81
HLA-B*35:01	307	315	LTSSSISFY	290.39
HLA-A*03:01	307	315		178.44
HLA-B*58:01	307	315		152.27
HLA-A*26:01	307	315		128.37
HLA-A*11:01	307	315		76.77
HLA-B*15:01	307	315		44.5
HLA-A*68:01	307	315		34.14
HLA-A*30:02	307	315		28.31
HLA-A*01:01	307	315		12.58
HLA-A*02:01	284	292	LVYAYHNLL	414.32
HLA-A*68:02	284	292		256.41
HLA-C*07:01	284	292		197.13
HLA-C*14:02	284	292		178.75
HLA-C*03:03	284	292		70.81
HLA-A*02:06	284	292		40.25
HLA-A*30:02	185	193	RMAFTFQTM	289.53
HLA-A*24:02	185	193		259.37
HLA-A*02:06	185	193		201.69
HLA-A*32:01	185	193		60.99
HLA-C*14:02	185	193		58.84
HLA-B*15:01	185	193		7.04
HLA-A*32:01	542	550	WVMFMFFYF	361.1
HLA-A*68:01	542	550		247.05
HLA-A*24:02	542	550		160.23
HLA-A*68:02	542	550		121.88

HLA-B*53:01	542	550		102.58
HLA-B*15:01	542	550		95.32
HLA-B*35:01	542	550		45.94
HLA-A*23:01	542	550		31.08
HLA-A*02:06	542	550		19.19

Table S6: MHC-I binding epitopes selected from hypothetical protein.

Allele	Start	End	Peptide	IC50
HLA-A*31:01	66	74	KVYEWDFSR	3.59
HLA-A*11:01	66	74		41.59
HLA-A*68:01	66	74		60.67
HLA-A*32:01	66	74		249.47
HLA-A*03:01	66	74		278.49
HLA-A*02:06	66	74		440.93
HLA-A*29:02	92	100	QMFNPPFVY	5.98
HLA-A*30:02	92	100		20.03
HLA-C*12:03	92	100		30.29
HLA-B*15:02	92	100		34.51
HLA-A*03:01	92	100		41.96
HLA-A*32:01	92	100		50.9
HLA-B*15:01	92	100		54.01
HLA-A*11:01	92	100		146.18
HLA-B*35:01	92	100		167.46
HLA-C*07:02	92	100		209.94
HLA-C*14:02	137	145	RLMNGHNSM	18.66
HLA-B*15:01	137	145		22.82
HLA-A*32:01	137	145		37.18
HLA-B*07:02	137	145		40.58
HLA-B*08:01	137	145		62.72
HLA-A*02:06	137	145		165.22
HLA-A*02:01	137	145		206.64
HLA-C*03:03	137	145		239.78
HLA-B*15:02	137	145		442.39
HLA-C*12:03	137	145		489.06
HLA-C*14:02	253	261	RYCCRRMVL	68.7
HLA-A*23:01	253	261		108.05
HLA-C*07:02	253	261		152.33
HLA-A*24:02	253	261		190.85
HLA-A*30:01	253	261		193.65
HLA-A*31:01	253	261		374.52
HLA-A*26:01	178	186	STIDPAQSY	11.38

HLA-A*29:02	178	186		32.66
HLA-B*15:01	178	186		54.46
HLA-A*30:02	178	186		81.44
HLA-A*11:01	178	186		102.29
HLA-C*14:02	178	186		237.57
HLA-A*25:01	178	186		313.5
HLA-C*12:03	178	186		333.42
HLA-B*15:02	178	186		484.91
HLA-A*29:02	232	240		6.07
HLA-A*01:01	232	240		34.95
HLA-B*35:01	232	240	YLSLLQAEY	117.32
HLA-B*15:01	232	240		171.02
HLA-A*30:02	232	240		346.36

Table S7: Ellipro analysis of discontinuous epitopes.

No.	Residues	Number of residues	Score
1	A:I357, A:N360, A:E361, A:N363, A:K364, A:F365, A:L366, A:A367, A:A368, A:A369, A:Y370, A:Q371, A:S372, A:I373, A:D374, A:L375, A:N376, A:E377, A:S378, A:N379, A:K380, A:F381, A:L382, A:A383, A:T384, A:A385, A:D386, A:D387, A:A388, A:A389, A:Y390, A:N391, A:P392, A:P393, A:F394, A:V395, A:Y396, A:S397, A:L398, A:A399, A:Y410, A:S411, A:L412, A:A413, A:I414, A:S415, A:T416, A:D417, A:G418, A:N419, A:W420, A:I421, A:A422, A:A423, A:Y424, A:D425, A:K426	57	0.801
2	A:H453, A:H454, A:H457, A:H458	4	0.765
3	A:M1, A:A2, A:K3, A:L4, A:S5, A:T6, A:D7, A:E8, A:L9, A:A12, A:M16, A:T17, A:L18, A:L19, A:E20, A:L21, A:S22, A:D23, A:F24, A:V25, A:K26, A:F28, A:E29, A:E30, A:T31, A:F32, A:V34, A:T35, A:A36, A:A37, A:V40, A:L67, A:E68, A:A69, A:A70, A:G71, A:D72, A:K73, A:K74, A:I75, A:G76, A:V77, A:I78, A:K79, A:V80, A:V81, A:R82, A:E83, A:I84, A:V85, A:S86, A:G87, A:L88, A:G89, A:L90, A:K91, A:E92, A:A93, A:K94, A:D95, A:L96, A:V97, A:A100, A:P101, A:L104, A:V108, A:A109, A:K110, A:E111, A:A112, A:A113, A:D114, A:E115, A:A116, A:K117, A:A118, A:K119, A:L120, A:E121, A:A122, A:A123, A:G124, A:A125, A:T126	84	0.722
4	A:S328, A:T329, A:I330, A:D331, A:P332, A:A333, A:Q334, A:S335, A:Y336, A:A337, A:A338, A:Y339, A:S340, A:I341, A:D342, A:L343, A:N344, A:E345, A:S346, A:N347	20	0.71
5	A:Y162, A:M163, A:G164, A:G165, A:P166, A:G167, A:P168, A:G169, A:H170, A:Q171, A:G172, A:H173, A:R174, A:M184, A:G185, A:P186, A:G187, A:P188, A:G189, A:Q190, A:G191, A:H192, A:R193, A:D195, A:P204, A:G205, A:P206, A:G207, A:P208, A:G209, A:G210, A:P211, A:G212, A:P213, A:G214, A:G215, A:H216, A:R217, A:F218, A:P228, A:K229, A:G230, A:P231, A:G232, A:P233, A:G234, A:F236, A:P246, A:K247, A:Y248	50	0.692
6	A:G145, A:P146, A:G147, A:P148, A:G149, A:K150	6	0.612

Table S8: Interacting residues of MHCI and vaccine.

No.	Receptor AA	Vaccine AA
1	ALA150	TYR162
2	ALA150	PRO186
3	ALA158	HIS450
4	ALA69	HIS456
5	ALA69	PHE175
6	ALA69	HIS456
7	ARG114	HIS455
8	ARG114	HIS457
9	ARG114	HIS458
10	ARG114	TRP147
11	ARG114	HIS457
12	ARG114	HIS458
13	ARG65	THR157
14	ARG65	PHE158
15	ARG65	PHE158
16	ASN66	GLN159
17	ASN66	TYR84
18	ASN66	ASN452
19	GLN70	HIS455
20	GLN70	HIS458
21	GLN72	ASP176
22	GLN72	HIS173
23	GLU152	TYR162
24	GLU152	HIS457
25	GLU153	HIS457
26	GLU166	LYS447
27	GLU166	TYR451
28	GLU58	PHE156
29	GLU63	HIS455
30	ILE97	HIS458
31	LEU156	HIS457
32	LYS146	PRO188
33	LYS146	PRO166

34	LYS146	GLY164
35	LYS146	GLY165
36	LYS146	PRO168
37	LYS146	PRO188
38	LYS68	GLN177
39	THR163	HIS450
40	THR163	TYR451
41	TYR159	HIS454
42	TYR159	HIS450
43	TYR84	PRO168
44	TYR84	PRO168
45	TYR99	HIS455
46	TYR99	HIS454
47	VAL76	HIS173
48	VAL76	HIS170

Table S9: Interacting residues of TLR2 and vaccine.

No.	Chain	Receptor AA	Atom	Vaccine AA	Atom
1	A	ARG296	NH1	ASP374	OD1
2	A	ARG296	NH1	GLU377	OE2
3	A	ARG296	NH2	ASP374	OD2
4	A	ARG321	HE	PRO253	O
5	A	ARG321	HH12	GLY283	O
6	A	ARG321	HH21	PRO253	O
7	A	ARG321	HH22	ARG282	O
8	A	ARG321	HH22	GLY283	O
9	A	ASP160	OD1	LYS447	HZ2
10	A	ASP160	OD2	LYS447	HZ3
11	A	ASP185	OD2	LYS151	HZ1
12	A	ASP185	OD1	LYS151	HZ2
13	A	LYS208	HZ1	ASP443	OD2
14	A	LYS208	H23	ASP443	OD1
15	A	MET159	O	LYS447	HZ1
16	A	PRO320		PRO284	
17	B	ASN414	OD1	ASP331	HN
18	B	ASP384	OD2	ARG321	NH1
19	B	ASP384	OD1	ARG321	NH2
20	B	ASP384	OD2	ARG322	NH1
21	B	CYS432	O	LYS229	HZ1
22	B	GLN433	HE22	LYS247	O
23	B	GLN433	OE1	LYS229	CE
24	B	GLU383	OE2	ARG321	HH21
25	B	GLU383	OE1	ARG322	HH22
26	B	GLU383	OE1	ARG322	HH22
27	B	GLU383	OE1	ARG322	NH1
28	B	GLU407	OE2	PRO251	CD
29	B	GLU407	O	PRO253	CD
30	B	GLU407	OE1	PRO253	CD
31	B	GLU436	HN	TYR248	
32	B	LYS378		PRO250	
33	B	LYS413	NZ	PHE249	

34	B	LYS413		ILE330	
35	B	LYS413		TYR248	
36	B	LYS437	HZ1	ASP331	OD2
37	B	LYS437	HN	TYR248	OH
38	C	ARG521	HH11	PRO188	O
39	C	ARG521	HH12	TYR162	OH
40	C	ARG521	HH22	TYR162	OH
41	C	ARG521	CD	PRO188	O
42	C	ARG521	O	PRO168	CD
43	C	ASP520	O	GLY167	HN
44	C	ASP520	OD1	HIS457	CE1
45	C	LYS551	HZ1	HIS457	O
46	C	LYS551	HZ1	HIS458	O
47	C	LYS551	HZ2	HIS457	
48	C	LYS551		HIS457	
49	C	PRO540	CA	HIS450	NE2
50	C	PRO540		PRO286	
51	C	PRO540		HIS450	
52	C	PRO575	O	HIS450	HD1
53	C	SER496	CB	PRO186	O
54	C	THR523	OG1	GLY167	CA
55	C	TYR544		HIS457	

Table S10: Interacting residues of GRP78 and vaccine.

No.	Chain	Receptor AA	Atom	Vaccine AA	Atom
1	A	GLY240	O	GLY167	HN
2	A	BAL241	CA	GLY167	O
3	A	PHE242	HN	GLY167	HN
4	A	ASP212	O	HIS457	CE1
5	A	ASN239	O	HIS457	HD1
6	B	GLU73	OE2	LYS151	HZ1
7	B	GLU73	OE1	LYS151	HZ2
8	B	GLU73	OE2	LYS151	HZ3
9	B	ARG49	HH11	ASP212	O
10	B	VAL50	HH12	HIS457	O
11	B	ARG60	HH11	TYR451	O
12	B	ARG60	HH11	HIS454	O
13	B	ARG60	HH21	HIS454	O
14	B	ASN389	HN	HIS458	NE2
15	B	TYR396	HH	HIS458	O
16	B	ASN59	OD1	TYR451	HH
17	B	ARG49	CD	HIS456	O
18	B	ARG49	CD	HIS457	O
19	B	ILE388	CA	HIS458	NE2
20	B	BAL50	O	HIS458	C
21	B	GLU392	OE1	HIS458	CD2
22	B	GLY387	O	HIS458	CE1
23	B	ARG60	NH	HIS451	
24	B	TRY396		HIS458	
25	B	ARG60		TYR451	
26	B	ILE388		HIS458	

Table S11: Discontinuous epitopes selected for both the proteins with the highest scores.

PROTEIN	DISCONTINUOUS EPITOPES	Residues	Score
Phytoene dehydrogenase	A:K512, A:I513, A:E514, A:M515, A:E516, A:N517, A:T518, A:Q519, A:A520, A:P521, A:L522, A:E523, A:E524, A:P525, A:D526, A:A527, A:E528, A:S529, A:T530, A:F531, A:P532, A:V533, A:W534, A:F535, A:W536, A:L537, A:R538, A:A539, A:A540, A:F541, A:W542, A:V543, A:M544, A:F547, A:F548, A:F551, A:P552, A:Q553, A:S554, A:N555, A:Q557, A:T558, A:P559, A:A560, A:S561, A:F562, A:I563, A:N564, A:N565, A:L566, A:L567, A:P568, A:E569, A:V570, A:F571, A:R572, A:V573, A:H574, A:N575, A:S576, A:N577, A:V578, A:I579	63	0.794
Hypothetical protein	A:S65, A:D82, A:N84, A:A85, A:E86, A:E87, A:P88, A:S89, A:A90, A:A91, A:Q92, A:M93, A:F94, A:N95, A:P96, A:P97, A:P125, A:P126, A:S127, A:K128, A:K129, A:S130, A:K131, A:T132, A:T133, A:K134, A:K135	27	0.735

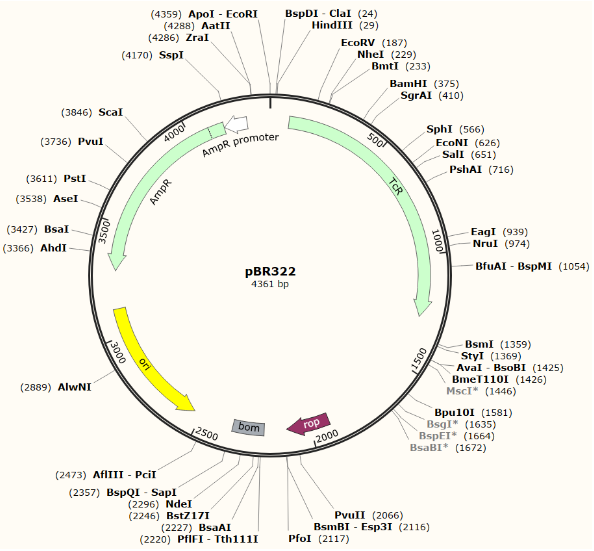

Figure S1: Sequence map of pBR322 utilized for cloning and expression.

hypothetical protein G6F55_004452 [Rhizopus delemar]	Rhizopus delemar
hypothetical protein G6F43_009965 [Rhizopus delemar]	Rhizopus delemar
hypothetical protein G6F23_006500 [Rhizopus oryzae]	Rhizopus oryzae
hypothetical protein G6F38_004552 [Rhizopus oryzae]	Rhizopus oryzae
phytoene dehydrogenase [Mucor lusitanicus]	Mucor lusitanicus
TerpK [Mucor circinelloides]	Mucor circinelloides
phytoene dehydrogenase [Mucor circinelloides 1006PhL]	Mucor circinelloides 1006PhL
phytoene dehydrogenase-like [Mucor ambiguus]	Mucor ambiguus
hypothetical protein INT46_004498 [Mucor plumbeus]	Mucor plumbeus

Figure S2: Blastp analysis of selected proteins for conservation among different fungal species.

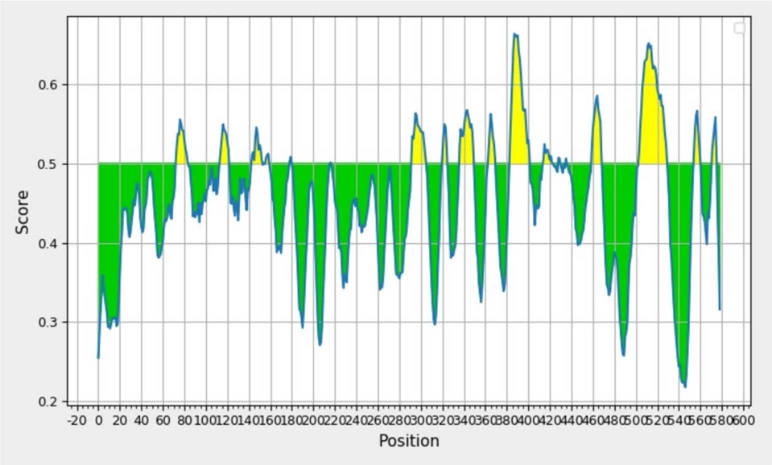

Figure S3: Predicted epitope scores for residues of phytoene dehydrogenase.

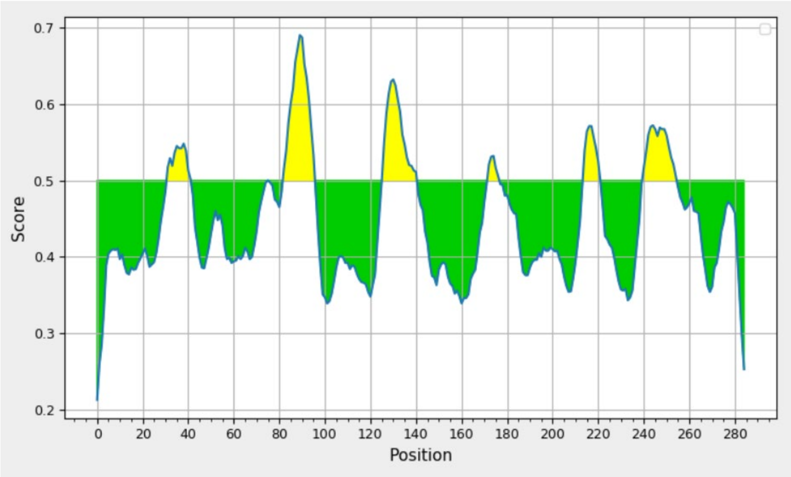

Figure S4: Predicted epitope scores for residues of hypothetical protein.

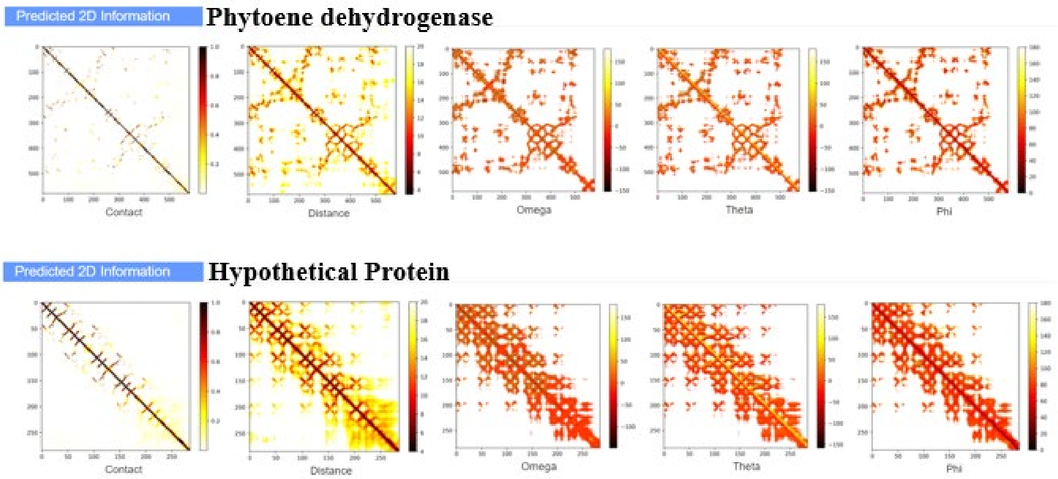

Figure S5: Contact maps and 2D information of both the target proteins.

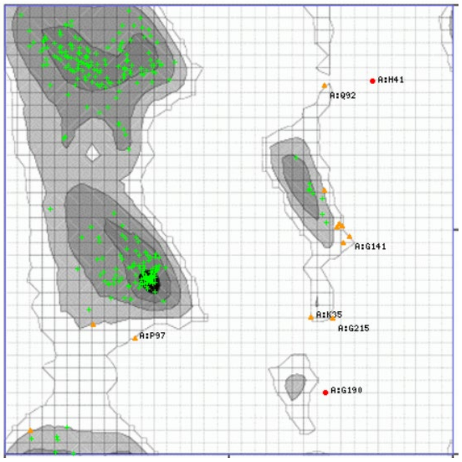

Figure S6: RC plot of the hypothetical protein secondary structure.

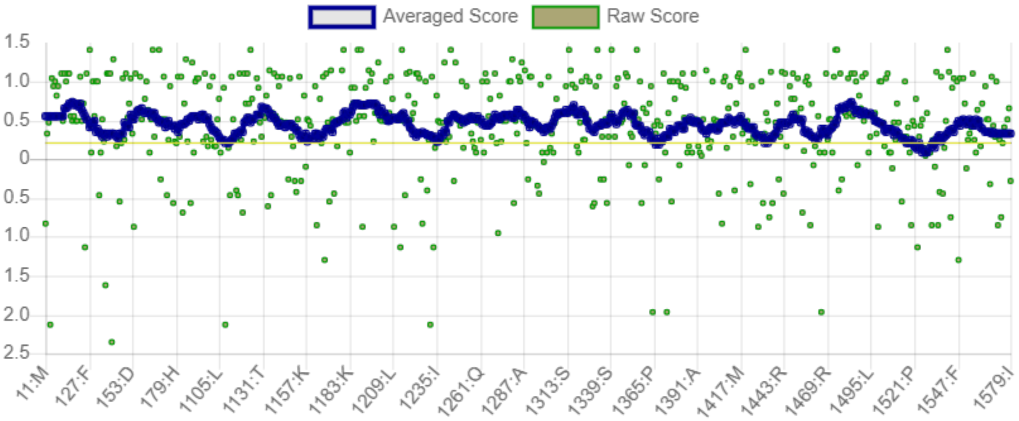

Figure S7: Verify3D plot of phytoene dehydrogenase.

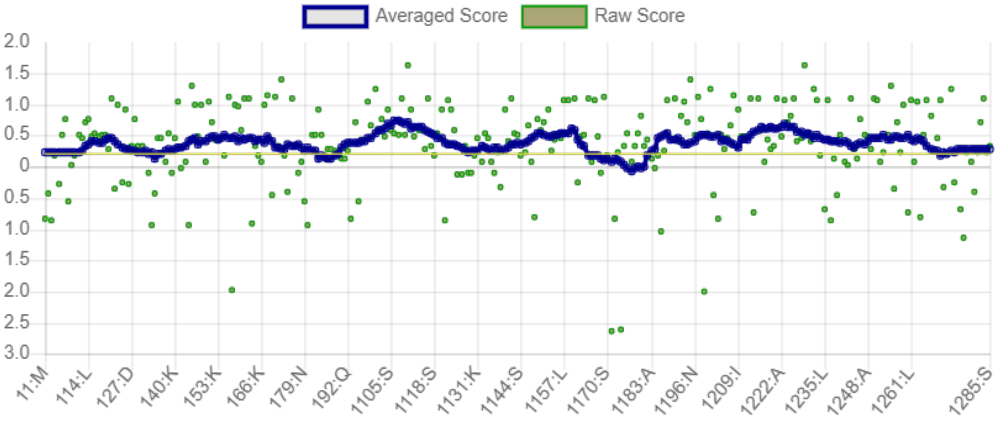

Figure S8: Verify3D plot of hypothetical protein residues.

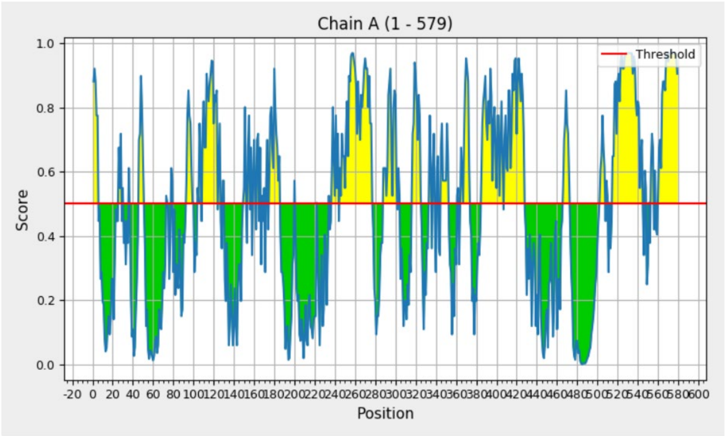

Figure S9: Ellipro epitopes graph for phytoene dehydrogenase.

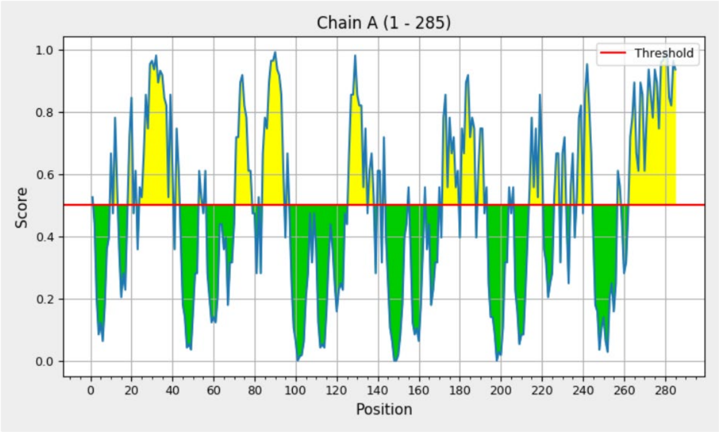

Figure S10: Ellipro epitopes graph for hypothetical protein.

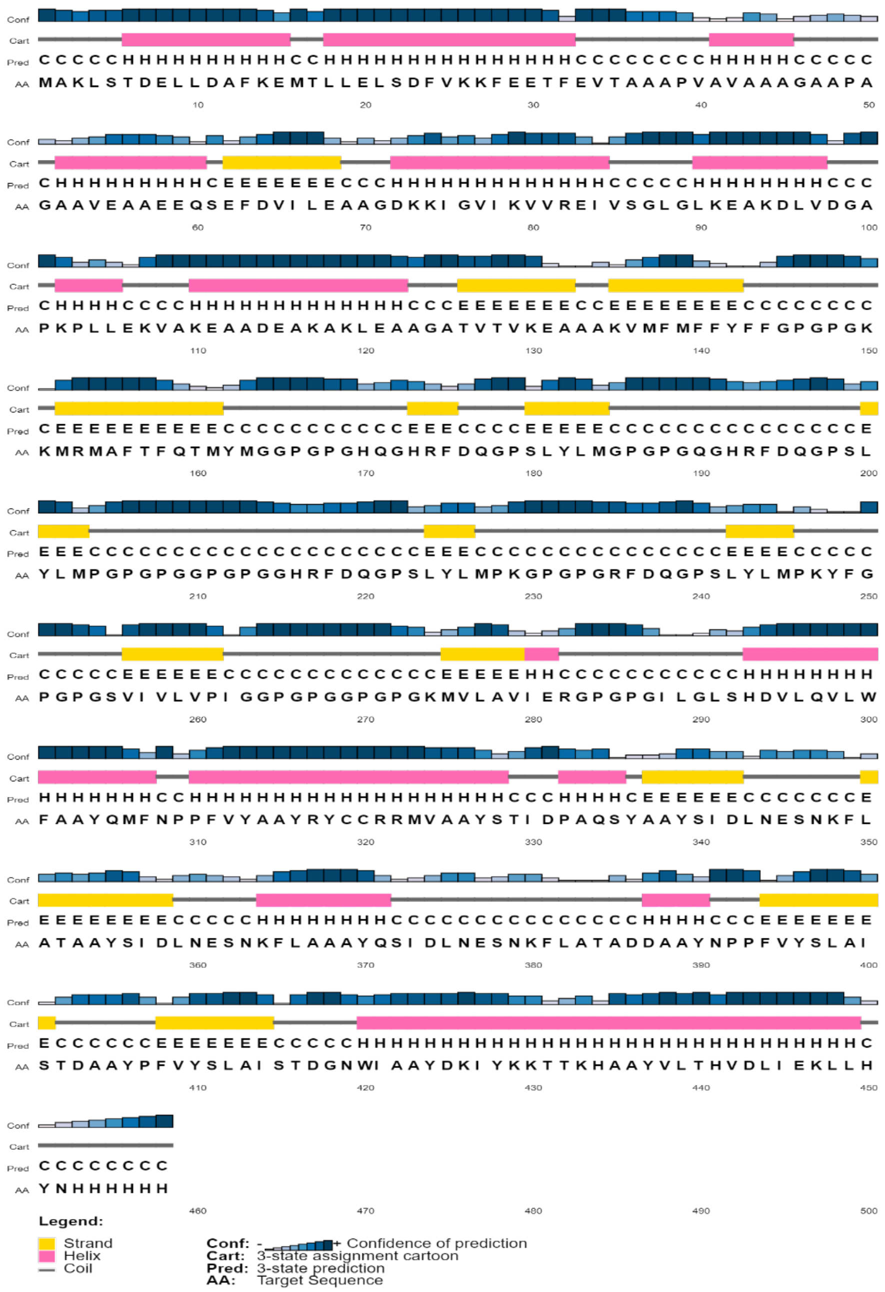

Figure S11: Cartoon analysis predicting the individual secondary structure of the vaccine.

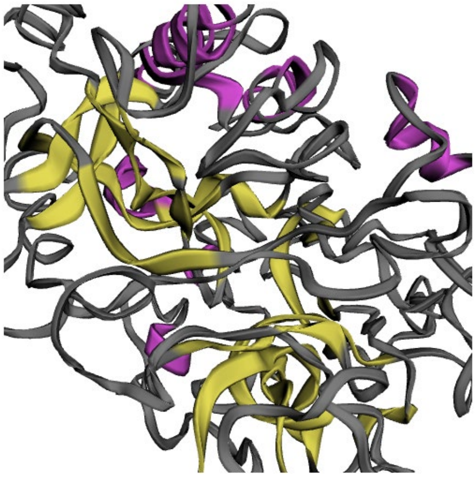

Figure S12: DPM fold structure of the vaccine construct.

```
Predicted Disorder Probability:
0.85 0.73 0.63 0.44 0.32 0.25 0.19 0.13 0.08 0.07 0.06 0.05 0.04 0.03 0.03 0.02 0.02 0.01 0.01 0.01 0.01 0.01 0.02 0.02 0.02 0.03 0.04 0.06 0.07 0.09 0.09 0.08
0.10 0.09 0.07 0.07 0.09 0.09 0.06 0.05 0.06 0.06 0.07 0.09 0.11 0.13 0.15 0.17 0.16 0.19 0.17 0.18 0.17 0.15 0.16 0.21 0.24 0.24 0.27 0.23 0.12 0.07 0.04 0.04
0.03 0.03 0.03 0.03 0.04 0.06 0.07 0.06 0.05 0.04 0.03 0.03 0.02 0.02 0.02 0.02 0.01 0.02 0.02 0.03 0.03 0.03 0.03 0.03 0.03 0.03 0.03 0.03 0.03 0.03 0.03 0.04
0.04 0.03 0.03 0.03 0.02 0.02 0.03 0.03 0.03 0.03 0.03 0.03 0.04 0.03 0.03 0.04 0.05 0.04 0.05 0.06 0.05 0.06 0.06 0.07 0.09 0.09 0.06 0.04 0.04 0.03 0.03 0.03
0.02 0.02 0.02 0.02 0.02 0.02 0.01 0.01 0.01 0.01 0.01 0.01 0.01 0.01 0.01 0.01 0.02 0.02 0.03 0.03 0.03 0.03 0.02 0.02 0.02 0.01 0.01 0.01 0.01 0.01 0.01 0.02
0.03 0.03 0.03 0.05 0.07 0.07 0.08 0.07 0.08 0.08 0.08 0.07 0.07 0.07 0.06 0.05 0.06 0.04 0.02 0.02 0.02 0.02 0.02 0.03 0.03 0.05 0.05 0.04 0.05 0.05 0.05 0.05
0.05 0.04 0.04 0.04 0.03 0.02 0.02 0.0
 2 0.01 0.02 0.02 0.03 0.05 0.07 0.09 0.08 0.12 0.11 0.12 0.13 0.13 0.16 0.14 0.13 0.14 0.15 0.16 0.18 0.09 0.04 0.03 0.03 0.02 0.03 0.04 0.03 0.05 0.07 0.07 0.09
0.07 0.10 0.11 0.10 0.08 0.08 0.05 0.03 0.02 0.02 0.01 0.01 0.01 0.01 0.02 0.02 0.02 0.02 0.02 0.02 0.01 0.02 0.01 0.01 0.01 0.01 0.01 0.01 0.01 0.02 0.02 0.04
0.05 0.07 0.06 0.08 0.07 0.07 0.07 0.05 0.04 0.03 0.02 0.01 0.01 0.01 0.01 0.01 0.01 0.02 0.02 0.02 0.02 0.02 0.03 0.02 0.02 0.02 0.02 0.02 0.02 0.02 0.01 0.01
0.01 0.01 0.01 0.01 0.01 0.01 0.01 0.01 0.01 0.01 0.01 0.01 0.01 0.01 0.01 0.01 0.01 0.01 0.01 0.01 0.01 0.01 0.01 0.01 0.01 0.02 0.02 0.02 0.02 0.03 0.02 0.02
0.03 0.03 0.03 0.04 0.03 0.03 0.02 0.02 0.02 0.02 0.01 0.01 0.01 0.02 0.03 0.03 0.04 0.03 0.03 0.03 0.02 0.02 0.02 0.01 0.01 0.01 0.01 0.02 0.02 0.02 0.03 0.03
0.04 0.03 0.04 0.03 0.03 0.03 0.03 0.02 0.03 0.02 0.04 0.04 0.04 0.05 0.07 0.06 0.07 0.06 0.06 0.05 0.06 0.07 0.07 0.05 0.07 0.06 0.06 0.05 0.04 0.03 0.03 0.02
0.02 0.01 0.01 0.01 0.01 0.01 0.01
 0.01 0.01 0.01 0.01 0.01 0.01 0.01 0.01 0.01 0.01 0.00 0.01 0.01 0.01 0.02 0.02 0.03 0.03 0.03 0.02 0.02 0.01 0.02 0.01 0.01 0.01 0.01 0.01 0.01 0.01 0.01 0.02
0.02 0.02 0.01 0.01 0.01 0.01 0.01 0.01 0.01 0.01 0.01 0.01 0.01 0.01 0.02 0.02 0.03 0.06 0.10 0.12 0.17 0.46 0.72 0.87 0.95 0.94 0.93
```

Figure S13: Disorder probabilities of the vaccine construct.

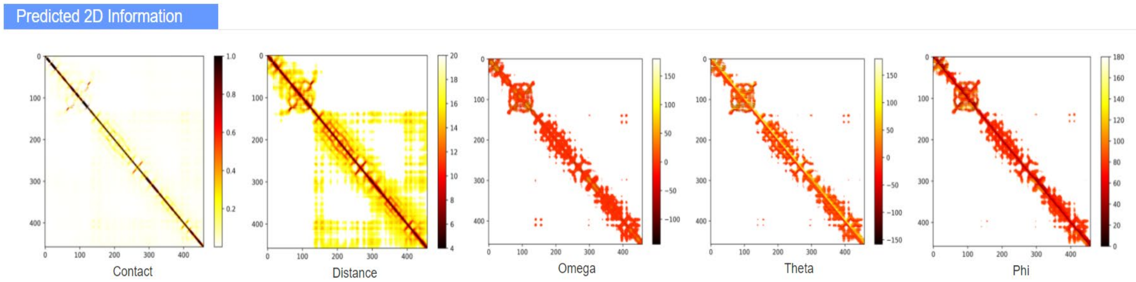

Figure S14: Contact maps of the 2D protein structure of the vaccine.

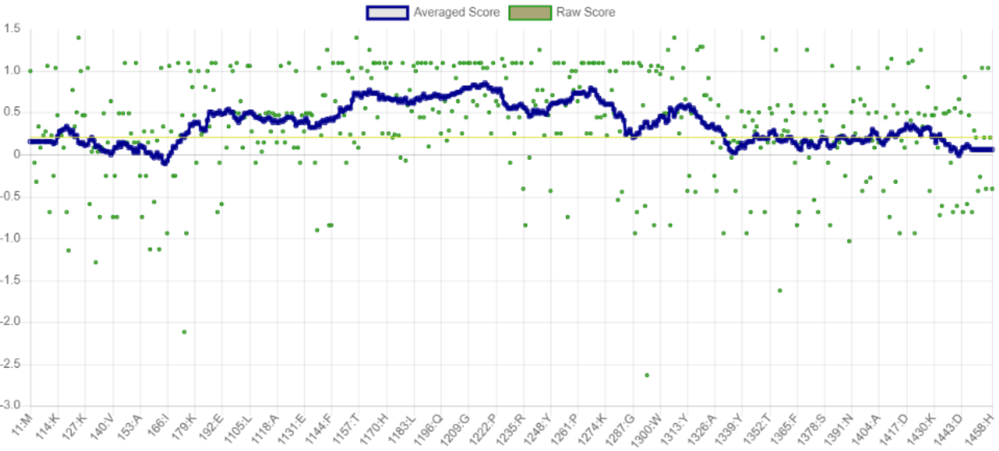

Figure S15: Verify3D plot of the vaccine construct.

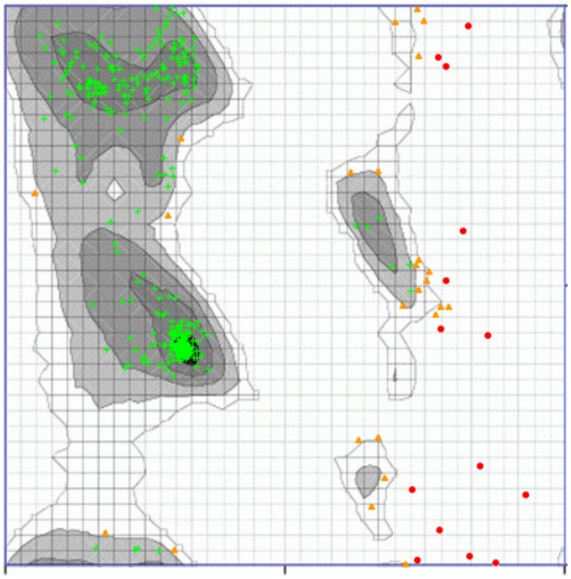

Figure S16: Confirmatory RC plot for the vaccine construct.

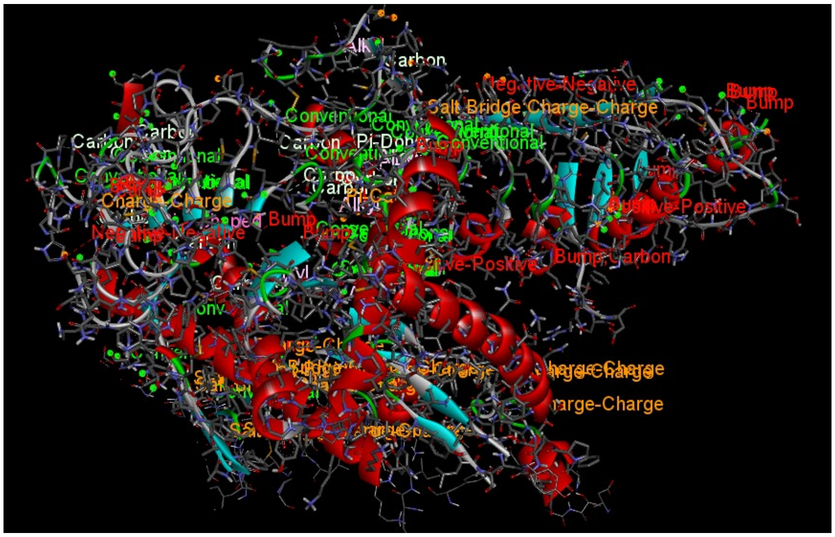

Figure S17: Interactions of TLR-2 with the vaccine.

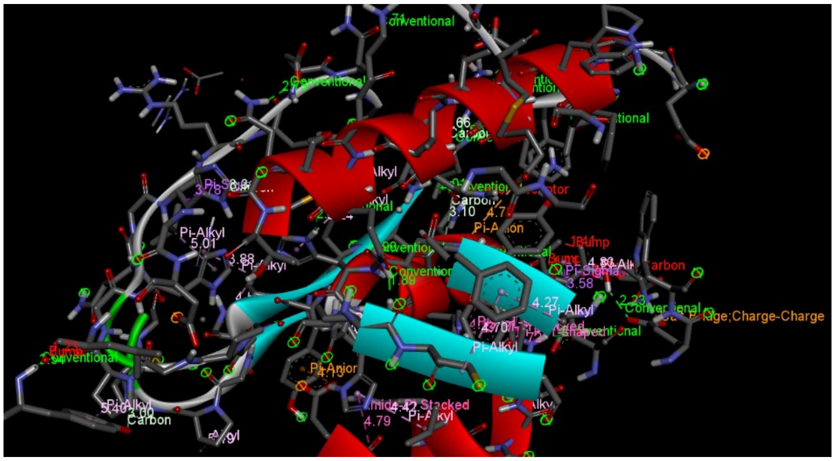

Figure S18: Interactions of MHC-I with the vaccine.

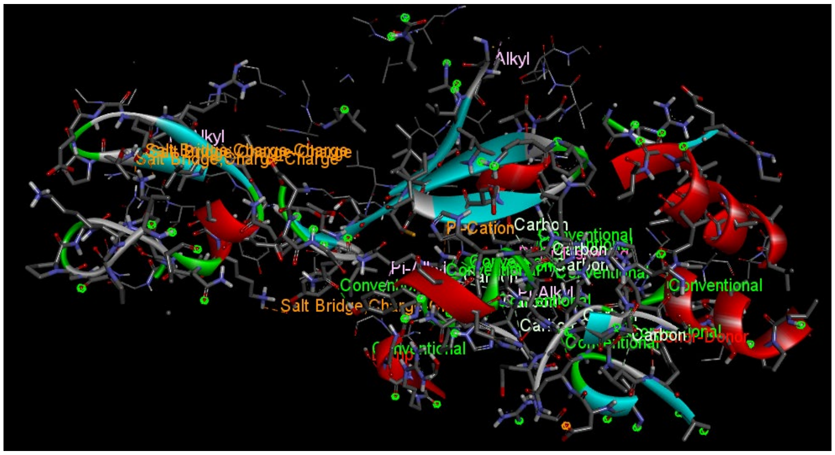

Figure S19: Interactions of GRP78 with the vaccine.

```
ATGGCCAAGCTGAGCACCGACGAGCTGCTGGACGCCTTCAAGGAGATGACCCTGCTGG
AGCTGAGCGACTTCGTGAAGAAGTTCGAGGAGACCTTCGAGGTGACCGCCGCCGCCCC
CGTGGCCGTGGCCGCCGCCGGCGCCGCCCCCGCCGGCGCCGCCGTGGAGGCCGCCGA
GGAGCAGAGCGAGTTCGACGTGATCCTGGAGGCCGCCGGCGACAAGAAGATCGGCGT
GATCAAGGTGGTGAGGGAGATCGTGAGCGGCCTGGGCCTGAAGGAGGCCAAGGACCT
GGTGGACGGCGCCCCCAAGCCCCTGCTGGAGAAGGTGGCCAAGGAGGCCGCCGACGA
GGCCAAGGCCAAGCTGGAGGCCGCCGGCGCCACCGTGACCGTGAAGGAGGCCGCCGC
CAAGGTGATGTTCATGTTCTTCTACTTCTTCGGCCCCGGCCCCGGCAAGAAGATGAGG
ATGGCCTTCACCTTCCAGACCATGTACATGGGCGGCCCCGGCCCCGGCCACCAGGGCC
ACAGGTTCGACCAGGGCCCCACCTGTACCTGATGGGCCCCGGCCCCGGCCAGGGCCA
CAGGTTCGACCAGGGCCCCAGCCTGTACCTGATGCCCGGCCCCGGCCCCGGCGGCCC
CGGCCCCGGCGGCCACAGGTTCGACCAGGGCCCCAGCCTGTACCTGATGCCCAAGGG
CCCCGGCCCCGGCAGGTTCGACCAGGGCCCCAGCCTGTACCTGATGCCCAAGTACTTC
GGCCCCGGCCCCGGCAGCGTGATCGTGCTGGTGCCCATCGGCGGCCCCGGCCCCGGC
GGCCCCGGCCCCGGCAAGATGGTGCTGGCCGTGATCGAGAGGGGCCCCGGCCCCGGC
ATCCTGGGCCTGAGCCACGACGTGCTGCAGGTGCTGTGGTTCGCCGCCTACCAGATGT
TCAACCCCCCCTTCGTGTACGCCGCCTACAGGTACTGCTGCAGGAGGATGGTGGCCGC
CTACAGCACCATCGACCCCGCCCAGAGCTACGCCGCCTACAGCATCGACCTGAACGAG
AGCAACAAGTTCCTGGCCACCGCCGCCTACAGCATCGACCTGAACGAGAGCAACAAGT
TCCTGGCCGCCGCCTACCAGAGCATCGACCTGAACGAGAGCAACAAGTTCCTGGCCAC
CGCCGACGACGCCGCCTACAACCCCCCCTTCGTGTACAGCCTGGCCATCAGCACCGAC
GCCGCCTACCCCTTCGTGTACAGCCTGGCCATCAGCACCGACGGCAACTGGATCGCCG
CCTACGACAAGATCTACAAGAAGACCACCAAGCACGCCGCCTACGTGCTGACCCACGT
GGACCTGATCGAGAAGCTGCTGCACTACAACCACCACCACCACCAC
```

Figure S20: The reverse translated sequence of vaccine construct.

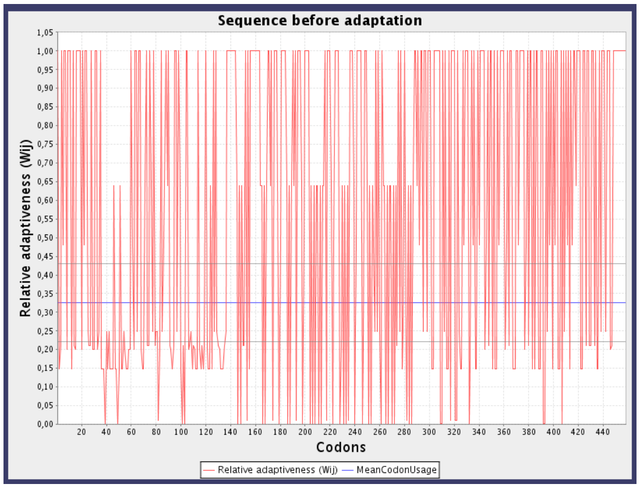

Figure S21: Codon adaptation pre-optimization.

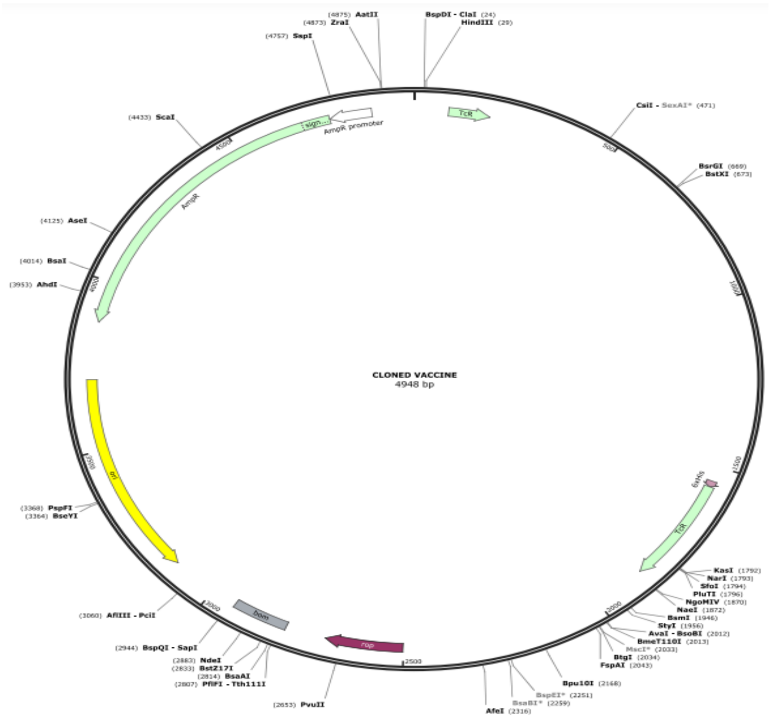

Figure S22: Cloned vaccine construct.

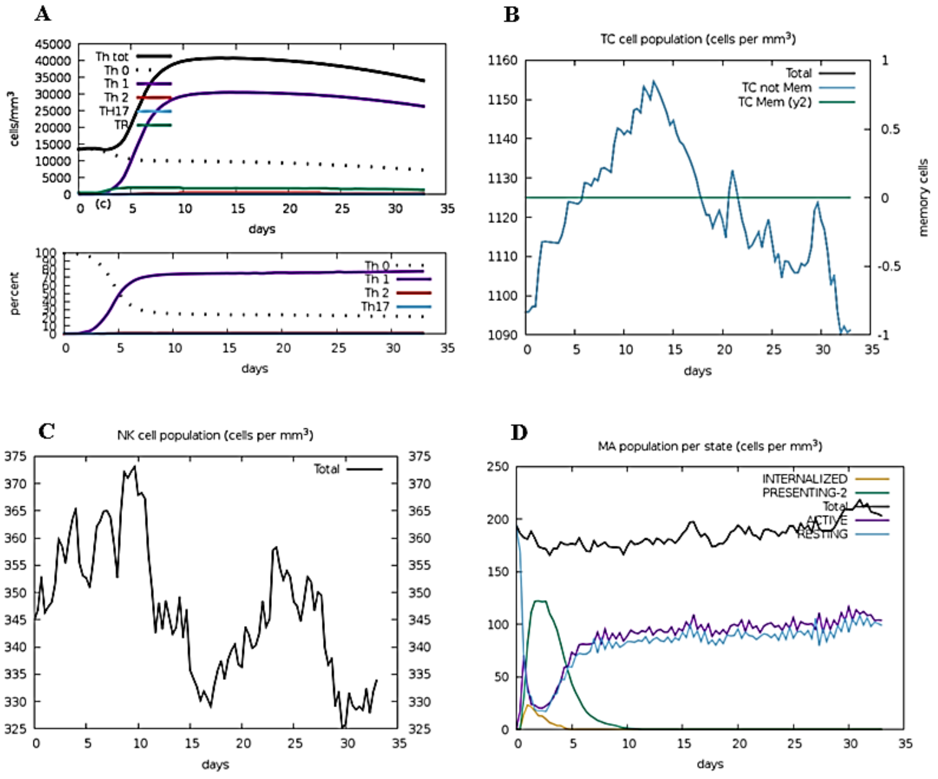

Figure S23: Innate and Adaptive immune response against vaccine injection.

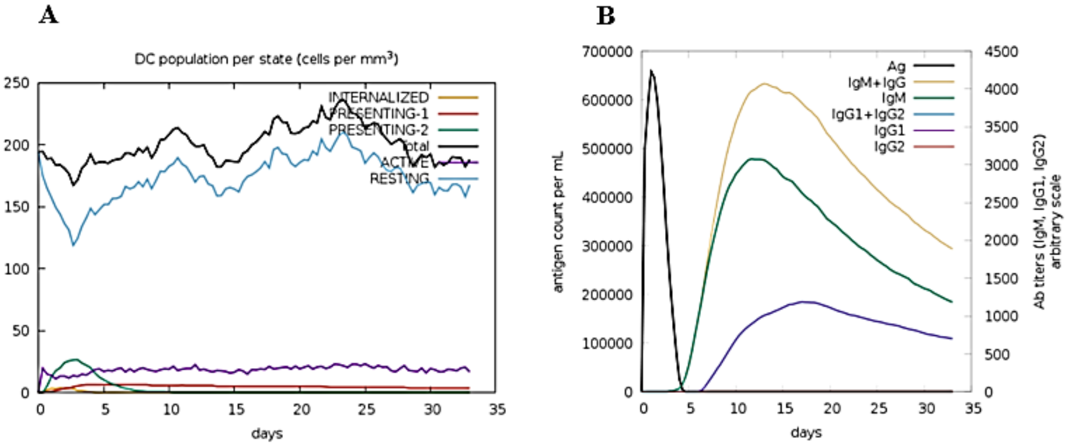

Figure S24: Immunocomplexes and DC response against the vaccine.

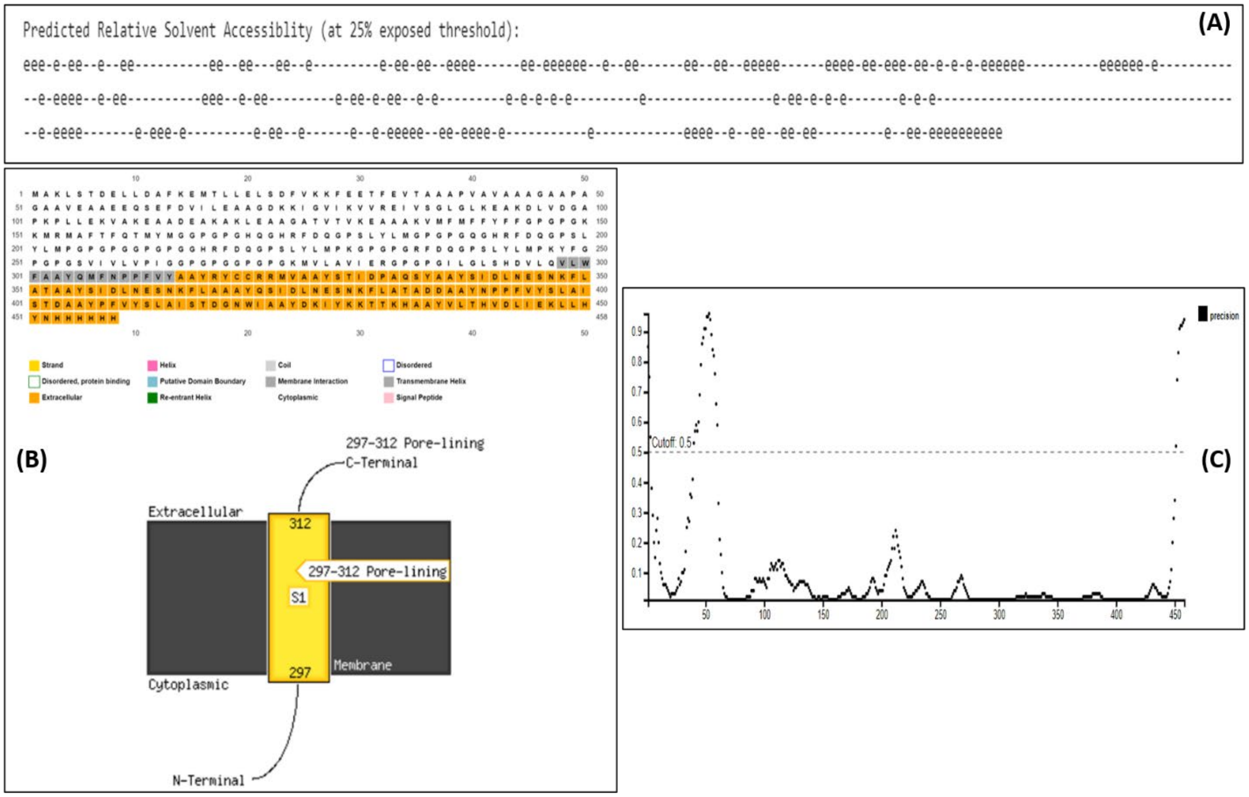

Figure S25: A) predicted solvent accessibility analysis of the vaccine construct. B) MEMSTAT analysis showing the subcellular location of the vaccine. C) DISOPRED plot showing the disordered regions.
